# Attentional bias and disengagement as a function of Body Mass Index in conditions that differ in anticipated reward

**DOI:** 10.1556/jba-9-818

**Published:** 2020-10-03

**Authors:** Afework Tsegaye, Joachim Bjørne, Anita Winther, Gyöngyi Kökönyei, Renáta Cserjési, H.N. Alexander Logemann

**Affiliations:** 1 Doctoral School of Psychology, ELTE Eötvös Loránd University, Budapest, Hungary; 2 Institute of Psychology, ELTE Eötvös Loránd University, Budapest, Hungary; 3 SE-NAP2 Genetic Brain Imaging Migraine Research Group, Hungarian Academy of Sciences, Semmelweis University, Budapest, Hungary; 4 Department of Pharmacodynamics, Faculty of Pharmacy, Semmelweis University, Budapest, Hungary; 5 Independent Researcher

**Keywords:** BMI, attentional bias, disengagement, reward, mindfulness

## Abstract

**Background and aims:**

Previous studies suggest that attentional bias and disengagement may vary as a function of Body Mass Index (BMI), most notably in a palatable food related context. Though this could indeed represent a food context specific effect, it could also represent a general reward related context effect. In addition, though mindfulness and stress have both been reported to affect attention, it is not yet clear whether these moderate the relationship between BMI and attention as a function of reward context. In the current study we addressed these questions. It was hypothesized that BMI would be positively associated with bias in a food context and money context relative to a neutral context. The inverse was expected for disengagement. It was expected that mindfulness would decrease these relationships and for stress the inverse was expected.

**Methods:**

In the current online study, eighty-seven participants (24 males and 63 females; age: *M* = 30.1, SD = 8.3; BMI: *M* = 24.2, SD = 4.67), filled out questionnaires and completed a visuospatial cueing task measuring attention and disengagement of attention in a neutral, food-related, and money-related condition.

**Results:**

There was no association between BMI and attentional bias. Higher BMI was associated with faster responses to money pictures presented opposite to a cued location as compared to money pictures that did not follow a predictive cue. Our results do not support a clear moderating role of mindfulness and stress.

**Discussion and conclusion:**

Our results imply faster processing and associated quicker responding to unanticipated reward-related stimuli in individuals with overweight or obesity.

## Introduction

Attentional bias and disengagement of attention are central to everyday functioning (Corbetta & Shulman, 2002). Attention is generally directed to relevant elements in our environment (attentional bias) resulting in facilitated processing of these elements. Stimuli can be relevant because they are salient, or they have relevance in relation to our current goals/tasks (Corbetta & Shulman, 2002). For instance, when individuals are hungry, the chance of consciously noticing stimuli related to palatable foods is relatively higher (Nederkoorn, Guerrieri, Havermans, Roefs, & Jansen, 2009). Of course, when we attend to a certain location in our environment, something relevant may occur at the unattended location. To be able to process information at that location, attention needs to be disengaged from the initial location and reoriented to the other, relevant location. This process of disengagement refers to the decoupling of attention allowing processing of initially unattended stimuli (Corbetta & Shulman, 2002). Although studies have suggested that weight, or more specifically Body Mass Index (BMI; kg/m^2^), is associated with attentional bias and disengagement, the exact relationship and role of (reward) context has not yet been elucidated.

To elaborate, consistent with the Incentive Sensitization theory (Robinson, Fischer, Ahuja, Lesser, & Maniates, 2016), results of previous studies strongly suggest that higher BMI is associated with enhanced motivation and attentional bias for food-related stimuli (Hendrikse et al., 2015; Volkow, Wise, & Baler, 2017; Yokum, Ng, & Stice, 2011). Attentional bias, in turn, predicts subsequent weight gain and persistence of excessive eating patterns (Yokum et al., 2011). Although studies on the relationship between BMI and disengagement of attention are lacking, several investigations have assessed the association between BMI and inhibitory control (Bartholdy, Dalton, O’Daly, Campbell, & Schmidt, 2016).

Inhibitory control can be defined as the ability to inhibit or abort a planned response, and is related to disengagement (Logemann et al., 2017). More specifically, both the process of inhibition and disengagement of attention are triggered by sudden changes in task-demands, and are driven by overlapping neurocircuitry (Logemann et al., 2017). Studies on the relationship between BMI and inhibitory control have yielded inconsistent results for neutral contexts (Bartholdy et al., 2016). However, in a recent study it was reported that higher BMI was associated with reduced inhibitory control in a palatable food context (operationalized using pictures representing palatable food) relative to a neutral context (operationalized using neutral letters), underscoring the importance of reward context (Houben, Nederkoorn, & Jansen, 2014). To the degree to which the process of inhibition overlaps with disengagement of attention, it may be expected that the aforementioned effect of BMI is mirrored with respect to disengagement. In other words, it may be expected that higher BMI is associated with reduced disengagement of attention from attended loci and/or stimuli that have reward value. In addition, previous studies suggest that rewards are processed differently in the brain (and specifically, the striatum) in individuals with obesity which resembles the pattern found in pharmacological addiction (Volkow et al., 2017). Hence, with respect to context, it seems possible that the aforementioned relationship is not specific to a food-context, but plausibly evident in any reward context.

With respect to other important moderators that may affect the relationship between BMI and visuospatial attention, studies suggest that stress may enhance the association between BMI and behavioral control (Tsegaye et al., 2020). Firstly, stress is known to induce preferential bias to palatable food (Oliver, Wardle, & Gibson, 2000), and palatable food may provide a negative reinforcement in a context of stress (Tsegaye et al., 2020). In other words, individuals that are stressed may ingest palatable food as a temporary escape from negative affect. Also noteworthy is the study of Nederkoorn, Smulders, Havermans, Roefs, and Jansen (2006). In their study, the relationship between BMI and inhibitory control was investigated using a stop signal task, which can be regarded as a relatively taxing (and plausibly relatively stressful) task. There was an inverse relationship between BMI and inhibitory control, but only at the end of the task. Hence, as stress may be induced as time on task increases, this may indicate that the relationship between BMI and inhibitory control varies as a function of (induced) stress.

A moderator that seems to affect the aforementioned relation in the opposite way, is mindfulness. There is some support for a direct relation between a mindful state and executive control. Specifically, enhanced mindfulness has been associated with generally improved attention (Sibalis et al., 2019), and improved inhibitory control (Gallant, 2016).

In the current study, we employed a Visuospatial Cueing (VSC) task, which is an ideal paradigm to measure attentional bias as well as disengagement (Posner, Snyder, & Davidson, 1980). In short, in the VSC task, a cue (i.e., arrow) signals the likely location of a subsequent target to which a response is required. Attentional bias is reflected in the speeded response times to validly cued targets relative to non-cued targets. Disengagement of attention is associated with reaction time costs and is reflected in the slowed responses to targets that appear at the non-indicated location (invalidly cued targets) relative to non-cued targets.

In the current study, we included three conditions, a neutral condition, a palatable food, and money condition. Targets in the neutral condition consisted of simple gray bars. Targets in the food condition represented palatable foods, similar to Houben et al. (2014). The novel money condition consisted of targets that represented money (which is generally rewarding).

It was hypothesized that higher BMI would be associated with increased attentional bias in both reward (food and money) contexts relative to the neutral context. Secondly, a negative relationship was expected between BMI and disengagement in the reward contexts relative to the neutral context. Finally, it was hypothesized that higher perceived stress would be associated with an increase of the aforementioned effects, and the opposite was expected for trait mindfulness.

## Methods

### Participants

Participants were included if they were between 18 and 50 years old, were not pregnant and had no known current mental disorder (by own admission), if they were not currently using drugs affecting cognitive functioning and had normal or corrected-to-normal vision. The final sample for the analyses consisted of in total eighty-seven individuals (24 males and 63 females; age: *M* = 30.1, SD = 8.3; body mass index (BMI): *M* = 24.2, SD = 4.67).

### Measures

#### Visuospatial Cueing (VSC) Task

**Fig. 1.**
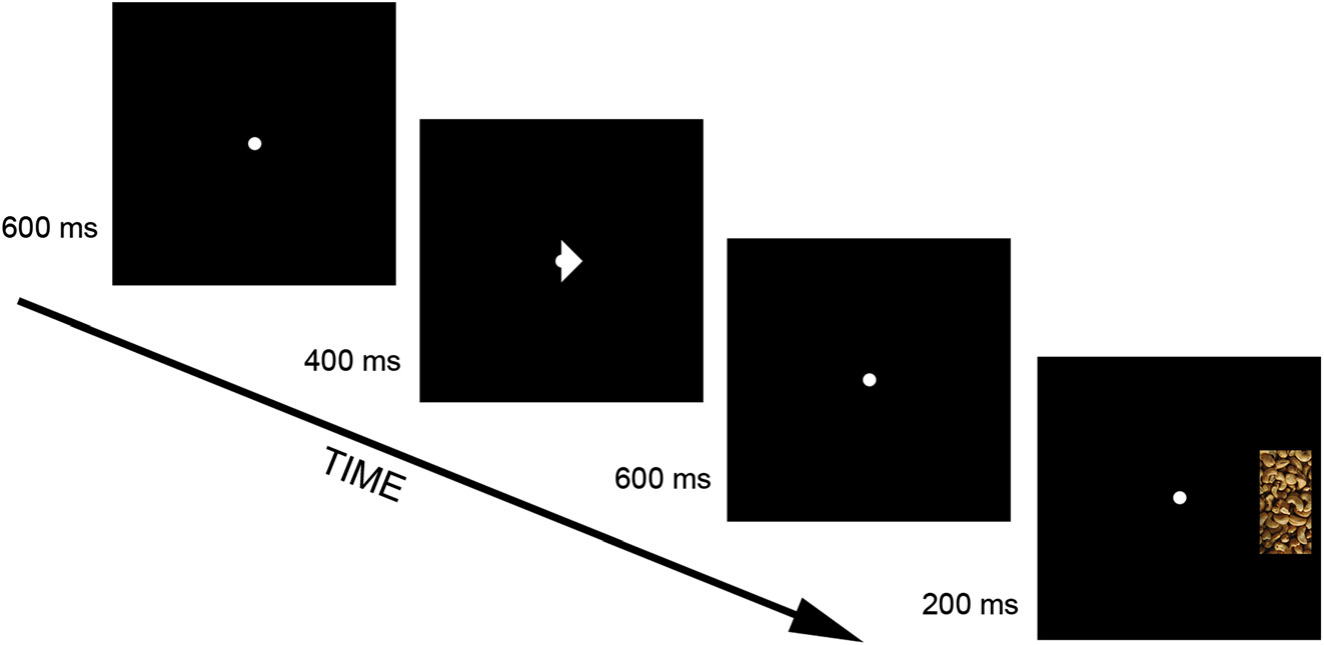
Graphical depiction of a valid trial in the food condition of the visuospatial cueing task

The VSC task was modeled after (Clark, Geffen, & Geffen, 1989; Logemann et al., 2017), and developed using Canvas for HTML5. The task was implemented on a Raspberry Pi3 with Linux operating system and Apache2 as webserver for online use. In a typical trial, a fixation dot was presented for 600 ms, after which a cue was presented centrally for 400 ms. The cue (width: 60 pixels; height: 60 pixels) indicated the likely location (unless in case of a non-informative cue) of a subsequent target to which a response was required. After presentation of the cue, the fixation dot was presented again for 600 ms after which the target was presented for 200 ms. The target was always a portrait-oriented bar-shaped stimulus, and presented at the vertical midline of the display, at the left or right side of the display. The required response depended on whether the bar (width: 200 pixels) was either long (height: 400 pixels) or short (height: 320 pixels). The current implementation included non-cued, invalid, and valid trials. In a non-cued trial, the cue was not informative of the location of the target. In invalid trials, the target was presented opposite to the location indicated by the cue. In valid trials, the target was presented on the side of the screen indicated by the cue (Fig. 1). The task consisted of three conditions, a neutral condition, a money, and food condition. The conditions differed only with respect to the target-pictures, the dimensions of the two (short and long) types of pictures did not differ between conditions. In the neutral condition, targets were solid gray filled bars. For the food condition, the short or long bar was always one out of four potential pictures representing palatable food (chips, chocolate, chocolate chip cookies, cashew nuts) similar to Houben et al. (2014). For the money condition, the short or long bar was always one out of four potential pictures representing money. Presentation of each picture, and type of target (short/long) was equiprobable. Condition order as well as response-target (short/long) assignment was counterbalanced over participants. Each condition consisted of 48 valid trials, 16 invalid trials, and 16 trials that consisted of non-informative cues. Trial order was randomized for each participant and trials could not be predicted from previous trials. Early (<150 ms) and late responses (>1,400 ms) were discarded from the analyses. The relevant outcomes of the VSC task were attentional bias, and disengagement. Bias was operationalized as the mean response time to non-cued targets minus the mean response time to validly cued targets. Disengagement was operationalized as the mean response time to invalidly cued targets minus the mean response time to non-cued targets. For both measures, the measurement level is milliseconds (ms).

#### Self-report measures implemented in Qualtrics (Qualtrics, Provo, UT, 2019)

##### Depression, Anxiety and Stress Scale (DASS-21) (Lovibond & Lovibond, 1995)

The DASS-21 consists of three self-report scales that is thought to measure the subjective degree of depression, anxiety, and stress. The subscales of DASS-21 have been reported to have a high reliability (Cronbach's *α* > 0.85) (Sinclair et al., 2012). In our sample (*N* = 87), the stress subscale also showed a good reliability (Cronbach's alpha: 0.891).

##### Mindful attention awareness scale (MAAS) (Brown & Ryan, 2003)

The MAAS is a 15-item scale that measures trait mindfulness. The questionnaire shows high internal consistency (Cronbach's *α* > 0.80) (Brown, West, Loverich, & Biegel, 2011), and in our sample (*N* = 87) Cronbach's alpha was 0.853.

### Procedure

The study was advertised on digital (social) media such as Facebook. The advertisement provided some brief information regarding the study, contact details of the investigator, and included a link to the online Qualtrics survey platform that included the information letter/informed consent form. After providing their informed consent, participants started with the questionnaires also implemented in Qualtrics. First, participants were requested to provide their age, gender, and hunger level. Hunger level was assessed with one question “Please state on a scale from 1(not hungry at all) to 5(very hungry) how hungry you currently are”. Subsequently, participants filled out the DASS-21 and MAAS. Upon completion, participants were directed to the online VSC task. For this task, participants were instructed to respond to the targets as fast and as accurately as possible. After performing the VSC task, the experiment was completed. The total duration of the experiment was approximately 30 minutes.

### Statistical analysis

Data preprocessing (to calculate the relevant outcome variables) was done using R (R Development Core Team, 2017) and statistical analyses were performed using SPSS (IBM Corp., 2019). Similar to Houben et al. (2014), we employed repeated measures ANCOVAs. Specifically, for evaluating the effects regarding attentional bias, we tested the BMI × condition (neutral/food) × validity (non-cued/valid) interaction with respect to response time (in ms level of measurement). In addition, we tested the BMI × condition (neutral/money) × validity (non-cued/valid) interaction with respect to response time. For disengagement, we performed the same tests, except the validity levels were non-cued/*invalid*. Lastly, we tested whether mindfulness and stress moderated the aforementioned interactions. Alpha was set at 0.05, and we controlled for age, gender, and hunger level. These variables were included in all analyses as covariates of no interest. All variables, except the within-subjects factor “condition” were continuous.

### Ethics

The study was approved by the Research Ethics Committee at ELTE Eötvös Loránd University, Faculty of Education and Psychology (reference number: 2017/218, date: 2017/10/25), and has been performed in accordance with the ethical standards as laid down in the 1964 Declaration of Helsinki and its later amendments or comparable ethical standards. Informed consent was obtained from all individual participants included in the study.

## Results

Descriptive data for age, BMI, mindfulness, stress, and hunger level are shown in Table 1. Data regarding the inferential statistics are reported in Table 2. Importantly, the well-known validity effect was replicated in the current study. Specifically, irrespective of condition, the validity effect was significant with shorter response times (RTs) to validly cued targets and longer RTs to invalidly cued targets relative to non-cued targets, *F*(1,86) = 109.61, *P* < 0.001 (partial *η*^2^ = 0.560), and *F*(1,86) = 6.37, *P* = 0.013 (partial *η*^2^ = 0.069) respectively.

**Table 1.** Descriptive statistics of age, BMI, mindfulness, stress and hunger level (*N* = 87)

	Max (maximum)	Min (minimum)	*M* (Mean)	SD (std. deviation)
Age	50.00	18.00	30.10	8.30
BMI	44.08	17.50	24.20	4.67
Mindfulness	6.00	1.93	4.06	0.74
Stress	40.00	0.00	13.10	9.05
Hunger level	5.00	2.00	4.18	0.84

**Table 2.** Primary analyses: attentional bias (top four rows) and disengagement (bottom four rows)

Factor	*F*(1,82)	*P*	partial *η*^2^
Condition (neutral/food) × validity (non-cued/valid)	9.77	0.002	0.106
BMI × condition (neutral/food) × validity (non-cued/valid)	2.37	0.128	0.028
Condition (neutral/money) × validity (non-cued l/valid)	6.53	0.012	0.074
BMI × condition (neutral/money) × validity (non-cued/valid)	2.53	0.116	0.030
Condition (neutral/food) × validity (non-cued/invalid)	8.23	0.005	0.091
BMI × condition (neutral/food) × validity (non-cued/invalid)	3.27	0.074	0.038
Condition (neutral/money) × validity (non-cued/invalid)	5.94	0.017	0.068
BMI × condition (neutral/money) × validity (non-cued/invalid)	5.95	0.017	0.068

*Note:* Dependent variable is response time in ms.

**Fig. 2.**
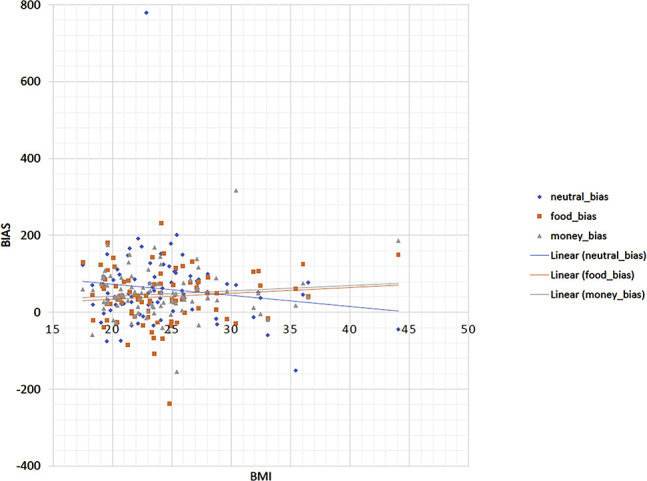
Graphical depiction of the relationship between BMI and bias (response time to non-cued targets minus validly cued targets, higher values indicate stronger bias) in the three conditions

**Fig. 3.**
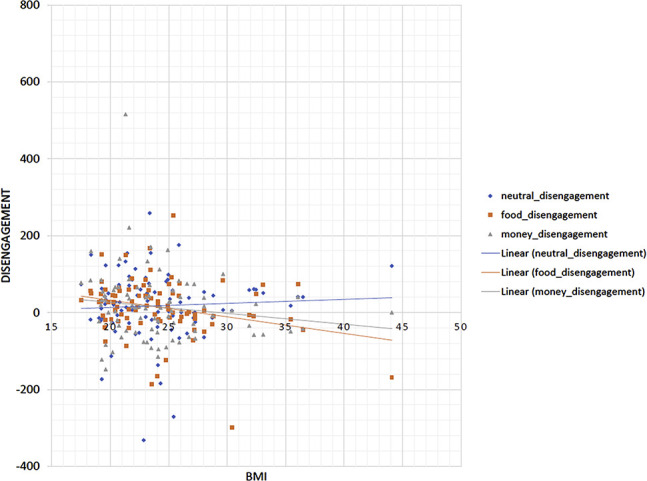
Graphical depiction of the relationship between BMI and disengagement (response time to invalidly cued targets minus non-cued targets, lower values indicate stronger disengagement) in the three conditions

Performance data with respect to bias and disengagement are graphically depicted in Fig. 2 and Fig. 3, respectively. Inferential statistics are shown in Table 2. As indicated by the condition (neutral/food) × validity (non-cued/valid) interaction, bias was reduced in the food condition relative to the neutral condition, but this effect was not affected by BMI. Similarly, as indicated by the condition (neutral/money) × validity (non-cued/valid) interaction, bias was significantly reduced in the money condition as compared to the neutral condition, and this effect was not affected by BMI.

As indicated by the condition (neutral/food) × validity (non-cued/invalid) interaction, disengagement was significantly enhanced in the food condition as opposed to the neutral condition and BMI did not affect this effect. Disengagement increased in the money condition compared to the neutral condition, as indicated by the condition (neutral/money) × validity (non-cued/invalid) interaction and BMI increased this effect. Posthoc testing indicated that higher BMI was associated with increased disengagement in the money condition (*F*(1,82) = 7.29, *P* = 0.008), but not in the neutral condition, F(1,82) = 0.95, *P* = 0.334.

With respect to the secondary explorative analyses, both self-reported mindfulness and stress did not affect any of the BMI × condition interactions that were described in Table 2 (all partial *η*^2^ < 0.023).

## Discussion

Previous studies have suggested that attentional bias may vary as a function of BMI. In addition, inhibitory control, and its associated process of attentional disengagement has also been implicated in relation to high BMI. However, the exact role of these aspects of visuospatial attention and the complex interaction between BMI with environment contexts of reward and subject variables (stress and trait mindfulness) had not yet been thoroughly investigated. Addressing this main gap in the literature, was the main aim of the current study.

The lack of a significant relationship between BMI and attentional bias to reward related stimuli relative to neutral stimuli was unexpected. Previous studies have suggested that high BMI (i.e., Yokum et al., 2011) and specifically obesity (Nijs, Muris, Euser, & Franken, 2010; Werthmann et al., 2011) is associated with increased attentional bias to food cues. However, it has also been noted that studies have yielded contradictory effects that may be accountable to differences in the exact methodology employed (Nijs & Franken, 2012). It is plausible that attentional bias for a specific location (as opposed to i.e., stimulus characteristics) is not affected by BMI.

With respect to disengagement, results are opposite to what was expected. Interestingly, higher BMI was associated with speeded responses to unexpected stimuli in the money condition (but not in the food condition) relative to the neutral condition. Results may imply that inhibitory control operates differently in the visuospatial cueing task as opposed to the stop signal task employed by Houben et al. (2014). Specifically, in the stop signal task the prepotent response to a stimulus that has reward value must be inhibited subsequent to a stop-stimulus. In this case, the requirement for inhibition contradicts with the primary task requirement (responding as fast and accurate as possible to the go stimuli). In the visuospatial cueing task however, disengagement of attention is congruent with the requirement to respond as fast and accurate as possible to the unattended target. In that vein, inhibition/disengagement may be speeded with increased perceived reward value of targets in the visuospatial cueing task, but negatively affected in the stop signal task (or go/no-go task).

It should be noted that there were some extreme values (defined as three times the interquartile range) with respect to bias (*n* = 2) and disengagement (*n* = 4). In view of the stringent exclusion criteria (erroneous responses were excluded, see materials) there is no reason to assume these represent erroneous data. Nevertheless, excluding the extreme cases did not change the significance of the BMI × condition (neutral/money) interaction with respect to disengagement.

With respect to the moderators, we did not find evidence for a moderating effect of stress on the aforementioned effects. This might seem to contrast previous studies on obesity that have suggested a moderating role of stress (Nederkoorn et al., 2006). However, we did not specifically focus on obesity, and we did not induce acute stress but assessed self-reported stress experience via the DASS-21 questionnaire which might not interact with BMI with respect to attentional/inhibitory control. Indeed, previous studies have shown a complex relation between stress and food intake. Although acute stress may affect food intake (Rutters, Nieuwenhuizen, Lemmens, Born, & Westerterp-Plantenga, 2009), a very recent study has suggested that the relationship between chronic stress and food intake may depend on the degree of impulsive-risk taking tendencies (Mason, Schleicher, Coccia, Epel, & Aschbacher, 2018).

Our results do not support a clear effect of trait mindfulness on the relationship between BMI and inhibitory control across the conditions. Previous studies have shown that mindfulness training improves executive control (Gallant, 2016; Sahdra et al., 2011), which in turn may promote weight loss. Indeed, with respect to obesity and weight-loss some systematic reviews have indicated that mindfulness-based training may promote weight loss (Carrière, Khoury, Günak, & Knäuper, 2018), but others have reported inconsistent findings (Rogers, Ferrari, Mosely, Lang, & Brennan, 2017; Ruffault et al., 2017). The main issue is that there are numerous mindfulness-based methods, which may differ in terms of rendered effects. In fact, a recent review suggests that specifically mindful eating, not mindfulness, is associated with subsequent weight loss (Dunn et al., 2018). To the best of our knowledge, the relation between mindfulness and executive mechanisms has not previously been thoroughly investigated in relation to BMI in contexts of reward.

The relationship between BMI and disengagement in the money condition was not evident in the food condition. One important difference between these conditions is the type of reinforcer. Food can be regarded as a primary reward/reinforcer whereas money becomes a secondary reinforcer via a conditioning process over time. Indeed, some differences exist with respect to the exact brain-regions that are activated subsequent to processing pictures of food and money, but both types of stimuli have been shown to activate the primary brain circuitry responsible for reward processing, and neural processing of these stimuli has been suggested to be modulated by metabolic state (Yousuf, Heldmann, Göttlich, Münte, & Doñamayor, 2018). The reason why the differential modulation of disengagement by BMI was limited for the contrast of the money versus neutral condition, might be due to the operationalization of the condition. Specifically, responses were more varied in the latter condition, negatively affecting statistical power. One limitation of the current study is that stimuli were not matched on salience. Thus, it might be that the enhanced variability in the food condition may be due to stimulus differences across conditions (i.e., salience).

Other limitations should also be noted. Firstly, height and weight were assessed by self-report, which can be affected by self-representation bias. However, as noted by Houben et al. (2014), it is not plausible that self-representation bias affects the overall rank-order of BMI values in the sample. Secondly, this was an online study. We should emphasize that studies suggest that the reliability of online cognitive experiments is comparable to those conducted in the lab (Hilbig, 2016). Most importantly, the overall validity effect was replicated in our study, confirming the validity of the employed paradigm. In a related vein, it has been suggested that the effects of cueing on performance is more substantial when perceptual demands and potential target locations are increased (Meinke, Thiel, & Fink, 2006). Indeed, in the current employed paradigm, participants plausibly quickly learned that there are only two possible target locations which might reduce the effect of cueing on response times. However, we should also emphasize again that the effect of cueing on response time was significant in the current paradigm and employment of a more taxing VSC task may result in a higher level of attrition. Lastly, although we controlled for gender, it should be noted that only four male participants had a BMI below the median. Excluding male participants, and performing the analyses on the female sample did not yield a different outcome (data available upon request). However, appropriate nuance should be applied in generalizing results to the male population.

In conclusion, our results suggest that higher BMI is associated with facilitated processing of unexpected stimuli that have general reward value. This might imply that individuals with overweight or obesity are sensitive to unexpected reward related stimuli and suggests that reward context should be considered in clinical context.

## Funding sources

GK was supported was supported by the MTA-SE-NAP B Genetic Brain Imaging Migraine Research Group, Hungarian Academy of Sciences, Semmelweis University (Grant No. KTIA_NAP_13-2-2015-0001); Hungarian Brain Research Program (Grant No. 2017-1.2.1-NKP-2017-00002). The preparation of this article was supported by the Hungarian National Research, Development and Innovation Office (Grant No. FK128614). RC was supported by Bolyai János Research Fellowship of the Hungarian Academy of Science.

## Authors' contribution

HNAL was the principal investigator. AT, GK, RC, and HNAL developed the project. JB wrote the code of the computer task and implemented it on the server. AW was responsible for participant-recruiting and data-acquisition under supervision of AT and HNAL. AT and HNAL performed the analyses. AT and HNAL developed the first draft of the manuscript. All authors had access to the source-data, interpreted the results and contributed to and approved the final article.

## Conflict of interest

The authors declare that there was no conflict of interest.
